# Development and testing of a standardized method to estimate honeydew production

**DOI:** 10.1371/journal.pone.0201845

**Published:** 2018-08-15

**Authors:** Melinda L. Moir, Michael Renton, Benjamin D. Hoffmann, Mei Chen Leng, Lori Lach

**Affiliations:** 1 School of Biological Sciences, University of Western Australia, Crawley, Western Australia, Australia; 2 Department of Primary Industries and Regional Development, South Perth, Western Australia, Australia; 3 School of Agriculture and Environment, University of Western Australia, Crawley, Western Australia, Australia; 4 CSIRO, Tropical Ecosystems Research Centre, Winnellie, Northern Territory, Australia; 5 Centre for Tropical Environmental and Sustainability Science, College of Science and Engineering, James Cook University, Cairns, Queensland, Australia; CSIRO, AUSTRALIA

## Abstract

Honeydew production by Hemiptera is an ecologically important process that facilitates mutualisms and increases nutrient cycling. Accurate estimates of the amount of honeydew available in a system are essential for quantifying food web dynamics, energy flow, and the potential growth of sooty mould that inhibits plant growth. Despite the importance of honeydew, there is no standardized method to estimate its production when intensive laboratory testing is not feasible. We developed two new models to predict honeydew production, one based on insect body mass and taxonomic family, and one based on body mass and life stage. We tested the accuracy of both models’ predictions for a diverse range of honeydew-producing hemipteran families (Aphididae, Pseudococcidae, Coccidae, Psyllidae, Aleyrodidae, Delphacidae, Cicadellidae). The method based on body mass and family provided more accurate estimates of honeydew production, due to large variation in honeydew production among families. We apply our methodology to a case study, the recalculation of honeydew available to invasive red imported fire ant (*Solenopsis invicta*) in the United States. We find that the amount of honeydew may be an order of magnitude lower than that previously estimated (2.16 versus 21.6 grams of honeydew per day) and discuss possible reasons for the difference. We anticipate that being able to estimate honeydew production based on minimal biological information will have applications to agriculture, invasion biology, forestry, and carbon farming.

## Introduction

Honeydew production by Hemiptera is an important ecological function in numerous systems globally. Many animals rely on honeydew as a food source, including birds [1], geckos [2], ants [3,4], bees [5], wasps [1,6], fruit flies [6], snails, cockroaches, moths [7], and hoverflies [8]. As an example of its importance as a food source, honeydew accounts for the majority of carbon brought into nests by wood ants, the nutrient cycling keystone species in boreal forests of Europe and Asia [9,10] and ant-hemipteran-honeydew associations are deemed ‘keystone interactions’ because of their importance to ecosystems [11]. Honeydew not consumed by animals promotes soil micro-organisms and fungal growth, and consequently, nutrient cycling [12,13,14,15]. The growth of sooty mould on honeydew has serious economic implications for some agricultural crops. For example, loss of viable kiwifruit in New Zealand can be as high as 85% from sooty mould caused by passionvine hopper (*Scolypopa australis* (Walker)) honeydew [16], losses of dates from the dubas bug (*Ommatissus lybicus* Bergevin) partly due to honeydew excretion can be up to 50% in the Middle East [17,18], and the invasive mealybug *Pseudococcus comstocki* (Kuwana) severely depreciates apples, pears, and peaches through honeydew-fuelled sooty mould outbreaks in Italy [19]. Hence, understanding the spatial and temporal distribution of honeydew production can provide important insight into food webs, ecosystem structure and function, and economic viability in agricultural systems.

Honeydew has been linked to the success of some invasive social insects. On Christmas Island, the invasive yellow crazy ant, *Anoplolepis gracilipes* Smith, and introduced honeydew-producing scale insects attain populations so high that they cause outbreaks of sooty mould, killing canopy trees [20]. In New Zealand, honeydew from native scale insects has facilitated a wasp invasion, resulting in the decline of the endangered kākā parrot (*Nestor meridionalis* (Gmelin)) through competition for honeydew [21]. Invasions of the red imported fire ant *Solenopsis invicta* Buren and Argentine ant *Linepithema humile* (Mayr) are facilitated by honeydew-producing Hemiptera [22,23,24,25]. Associations between honeydew-producers and mutualist partners can be so intertwined that when one is removed, the other disappears [26,27].

Despite the potential utility of accurately estimating honeydew production for both practical and theoretical purposes in a range of disciplines, a simple standardized method has not yet been developed. Accurate honeydew quantification is very difficult because honeydew production rates differ among life stages within a honeydew-producing species [25,28,29], across different host plants [25,30], with time of day [31], and the amount of nutrients that the host plant is receiving [32]. Quantification of honeydew production is predominantly achieved through time-intensive laboratory studies, and has been limited to a few economically important pest species [25,28,33,34,35,36,37]. But such intensive research is often unfeasible, particularly when dealing with diverse native faunas. Even when dealing with single species, their ecological requirements (e.g., host plant species, humidity, temperature, essential mutualists) and niche limits must be known before laboratory experiments can begin [38] (e.g., Perdikis & Lykouressis 2004). Alternatively, honeydew has been estimated by using traps or collection pans under plants infested with Hemiptera [15,39]. There are a number of problems with this collection method, however, including the influence of trap placement on the amount of honeydew collected, and the inability to determine how much honeydew has already been harvested by other taxa. In addition, honeydew traps do not discriminate among honeydew-producing species or account for differences produced by different life stages of Hemiptera. Thus, honeydew production has never been quantified for most species.

To help resolve problems with quantifying the amounts of honeydew produced by different hemipterans, we tested whether we could achieve accurate estimates of honeydew production based on biological traits and taxonomy. We developed two new methods of honeydew quantification, one based on taxonomic family identity and size of the hemipteran, and the second on life stage and size, and tested the ability of these methods to predict honeydew production for independent data through cross-validation.

## Materials and methods

Research on invertebrates does not require animal ethics approval in Western Australia. Experimental field work was covered by Department of Parks & Wildlife fauna licence SF009850.

### Published data

In June 2015 we searched the databases Google Scholar and Web of Science using the keywords: ‘honeydew’ and ‘production’, in combination with each of the following, ‘Coccidae’, ‘Coelostomidiidae’, ‘Pseudococcidae’, ‘Aphididae’, ‘Hemiptera’, ‘Aleyrodidae’, ‘Delphacidae’, ‘Membracidae’, ‘Kerriidae’, ‘Eurymelidae’, and ‘Psyllidae’. Of the 244 papers found, we identified 29 papers that quantified rate of honeydew production per individual, and specified species identity, lifecycle stage, and wet body mass (Table 1; S1 Appendix). When papers were data deficient, we contacted the authors for the necessary information.

**Table 1:** Collation of honeydew production in adults of different hemipteran species (except when adult data was not available). Additional details including honeydew produced by different nymphal stages, attending ants, host plants, variation (standard error) in honeydew production and source of data within each reference is given in S1 Appendix when available.

Family	*Species*	Life stage	Mean Honeydew produced (μg/hr)	Body mass (μg)	Honeydew References
Aleyrodidae	*Bemisia tabaci*	Adult ♀	2.66	51	[40]
Aphididae	*Aphis fabae*	Adult	40–151.5	930–2140	[30,41,42]
	*Aphis gossypii*	Adult	3.71	1112.45	[35, 43]
	*Aphis craccivora*	Adult	9.55	104.5	Moir et al.
	*Acyrthosiphon kondoi*	Adult	22.14	351.75	Moir et al.
	*Acyrthosiphon pisum*	Adult	61.25–87.48	3810–4086	[28] Moir et al.
	*Brachycaudus cardui*	Adult	190	2280	[42]
	*Lipaphis pseudobrassicae*	Adult	43.75	988.6	Moir et al.
	*Macrosiphoniella tanacetaria*	Adult	40	3574	[42]
	*Macrosiphum pisi*	Adult	44.28	2169	[36]
	Metopeurum fuscoviride*	Adult	435	1487	[44]
	*Metopolophium dirhodum*	Adult	18–23.85	2440.80	[45,46]
	*Neotoxoptera formosana*	Adult	13.69	902.85	Moir et al.
	*Rhopalosiphum padi*	Adult	10.35–27.96	148.5–398	[46] Moir et al.
	*Sitobion avenae*	Adult	20.20	1400	[45]
	*Therioaphis maculata*	Adult	44.71	464	[36]
	*Toxoptera aurantii*	Adult	53.27	2440.8	[33]
	Tuberolachnus salignus*	Adult	750–2048.4	12000–13860	[47,48,49]
	*Uroleucon sonchi*	Adult	149.33	3505.4	Moir et al.
Cicadellidae	*Cicadulina mbila*	Adult	41.25–254.58	500–1040	[50]
	*Nephotettix cincticeps*	Adult	1041.67–3744	2330^	[51,52]
	*Nephotettix virescens*	1^st^ and 5th	40.25–306.81	87.09–2100	[53]
	*Dalbulus quinquenotatus*	Adult	16203.7	2954.18	[54]
	*Dalbulus maidis*	Adult	4629.63	2423.83	[54]
	*Dalbulus gelbus*	Adult	2314.81	1975.97	[54]
Coccidae	*Coccus hesperidum*	Adult	284	2927.20	[55]
Delphacidae	*Nilaparvata lugens*	Adult	208.33–875	1390–2330	[37,53,56,57]
	*Sogatella furcifera*	Adult	354.70	1600	[29]
Membracidae	*Guayaquila xiphias*	Adult	7833–13500	51200	[58]
Pseudococcidae	*Phenacoccus solenopsis*	Adult	113.51–169.08	3574.9–4343.5	[25]
	*Dysmicoccus neobrevipes*	Adult	0.83	2224.04	[59]
	*Planococcus ficus*	2^nd^ instar	1.18	119.34^	[34]
	*Pseudococcus viburni*	2^nd^ instar	1.16	119.34^	[34]
	*Pseudococcus longispinus*	2^nd^ instar	0.72	119.34^	[34]
Psyllidae	Plant louse (*Acizzia* sp. nov.)	Adult	12.18	322.37	Moir et al.

*Renowned high producers of honeydew;

^estimate based on other comparable species; Moir et al. indicates this study

Our calculations required wet honeydew weight in μg as a function of body mass. In papers where honeydew volume was recorded, we multiplied the volume by the specific gravity of honeydew, 1.04 [41] to obtain the weight and then converted to μg. For six hemipteran species for which we had honeydew production data (*Toxoptera aurantii* (Fonscolombe), *Coccus hesperidum* Linnaeus, *Dysmicoccus neobrevipes* Beardsley, *Planococcus ficus* (Signoret), *Pseudococcus viburni* (Signoret) and *Pseudococcus longispinus* (Targioni)) we were unable to obtain the body mass either through published accounts, contacting other researchers, or sourcing live specimens ourselves. Instead, we calculated body mass from dry weight. To do this for all hemipteran species, we used as a model the body mass and lengths of *Phenacoccus solanopsis* Tinsley instars and adults [25,60] because this was the only hemipteran species with published records. We then used the power model of Gruner [61] to obtain dry weights of *P*. *solanopsis*. The power model *y* = *ax*^*b*^ uses standard coefficients for (a) and (b), length in millimeters (x), and dry mass in grams (y) ([61] and S2 Appendix for a and b coefficients). The known body mass of each *P*. *solanopsis* instar was then divided by its dry weight to obtain the multiplication factors needed for each instar; 1 for 1^st^ instars, 1.61 for 2^nd^ instars, 2.13 for 3^rd^ instars, and 6.11 for 4^th^ instars and adults. To our knowledge, no published information exists on the specific coefficients for the families Membracidae, Pseudococcidae, Coccidae, Margarodidae and Aleyrodidae. We therefore used the closest phylogenetic family as a proxy for these families; Cicadellidae for Membracidae, and Aphididae for Pseudococcidae, Coccidae, Margarodidae and Aleyrodidae (S2 Appendix).

We obtained honeydew production rates for 24 species representing 6 families from the literature (see S1 Appendix). The different feeding mechanisms of Hemiptera (e.g., phloem, xylem, parenchyma) often dictate the magnitude of honeydew produced, thus we ensured that we incorporated representatives from as many feeding strategies as possible.

### Experimental trials

To supplement data available in the literature, we conducted trials on seven locally available hemipteran species from two families (S1 Appendix) using a mixture of laboratory and field conditions in Perth, Western Australia (31°56’ S, 115°51’ E). We used potted plants in the laboratory trials, and naturally growing plants in the field, and in both cases the Hemiptera were feeding on stems or leaves. Sampling followed the protocols used by prior research [62,63]. A pre-weighed parafilm bag was placed over a hemipteran feeding on the plant, and the excreted honeydew accumulated within the bag. We assessed five replicates of each life stage, per hemipteran species. An additional five bags that did not contain any Hemiptera served as controls for plant evapotranspiration. In field conditions, we covered the parafilm bags with a light cloth during the day to prevent excessive evapotranspiration. We repeated trials when insects did not reattach to the host plant after being disturbed during bagging, Hemiptera escaped the bags, or evapotranspiration rates were high.

After 24 hrs we removed the bags, within which we gently dislodged the hemipterans with a fine paintbrush. In the lab, hemipterans were removed from bags, and the bag weighed on a Sartorius ISO 9001 precision scale. The Hemiptera were weighed separately on a Mettler Toledo XP6 scale to obtain their body mass. To obtain the honeydew produced in 24 hours, we subtracted the pre-weighed bag weight plus any moisture in control bags, from the total bag plus honeydew weight. We divided the honeydew weights by the number of Hemiptera within that life stage and species to obtain honeydew rates per individual hemipteran.

### Standardized methods to predict honeydew production

We developed and tested two possible methods to predict honeydew production depending upon the information available. The first method requires body mass and family of the hemipteran, and the second method requires identification of the life stage and body mass.

#### Honeydew prediction Method 1: Family identity and body mass

**Fig 1:**
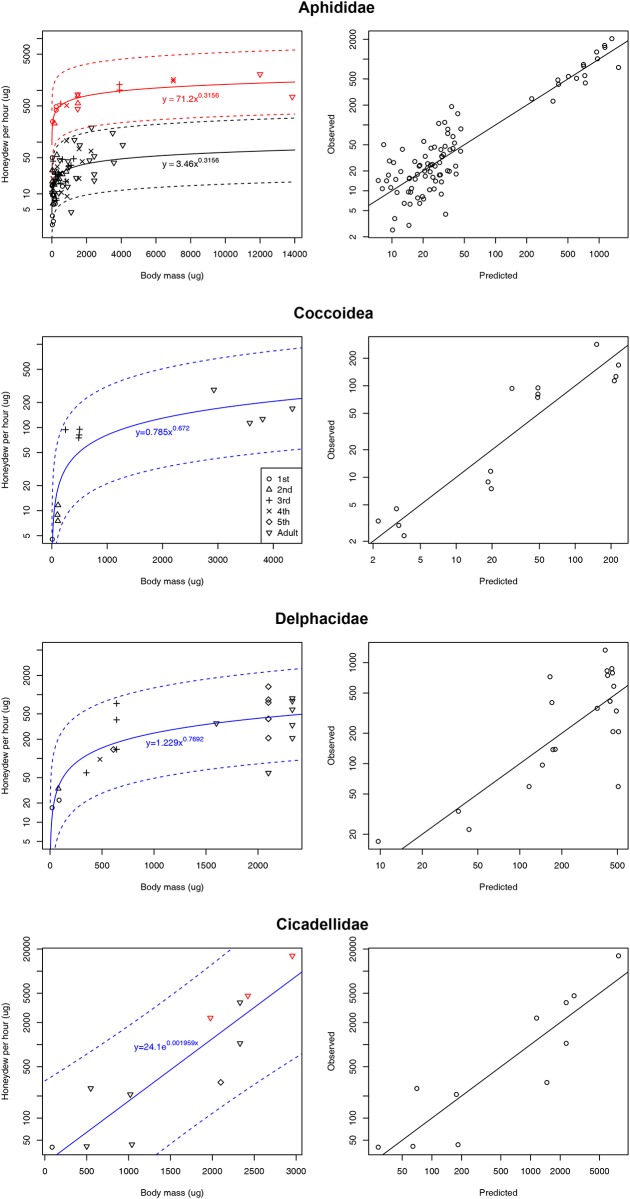
Empirical data for honeydew production per hour (on log scale), together with the model of best fit, for Aphididae (power-law), Coccoidea (power-law), Delphacidae (power-law), and Cicadellidae (exponential), showing actual values (symbols), model prediction (solid line) and 95% prediction interval (dashed lines). Symbols of life stage are as follows: circle—1^st^ instar nymphs, triangle—2^nd^ instar, plus symbol—3^rd^ instar, cross—4^th^ instar, diamond—5^th^ instar, upside down triangle—adults. Also shown for each family is the results of model validation: observed against independently predicted values. The best model for Aphididae used a different intercept term for the excessively high producing aphids *Tuberolachnus salignus* and *Metopeurum fuscoviride* (shown in red). Similarly the best model for Coccoidea omitted the extremely low producer *Dysmicoccus neobrevipes* (not shown).

Method 1 predicts honeydew production over 24 hours (y) as a function of body mass in μg (x) for a given family. We considered two models for each family: the power model (*y* = *ax*^*b*^) and exponential model (*y* = *a*e^*bx*^), and also considered whether either of these was better than a null model. To determine the values of the parameters *a* and *b* in the first two models we used linear regression in R [64], with log-transformed y data for the exponential model and log-transformed x and y data for the power model. Note that the power model is equivalent to a log-log linear model log(*y*) = *a*+*b*.log(*x*). Model comparison was based on AIC, where lower AIC indicates a better model. Although a common pattern between log honeydew and body mass was evident within most hemipteran families that we assessed, there were some exceptions. Two aphid species were extraordinarily high honeydew producers; *Tuberolachnus salignus* Gmelin and *Metopeurum fuscoviride* Stroyan. The latter produces honeydew at an hourly rate of 630% of its body mass as a 1^st^ instar and 30–55% as an adult [42,44]. We excluded these species from our models because their exceptional honeydew production rates are already well-documented, contributing towards their status as economically important pests. Nonetheless we included these high producers in plots (Fig 1) to illustrate their potential for honeydew production relative to other Hemiptera. Other pest species, including the aphids *Aphis fabae* Scopoli and *Acyrthosiphon pisum* (Harris) did conform to their respective family’s standard model.

#### Honeydew prediction Method 2: Life stage and body mass

Using this method, honeydew production (y) as a percentage of hemipteran body mass (μg) is estimated as a function of the life stage (x) represented as 1 for 1^st^ instar, 2 for 2^nd^ instar, 3 for 3^rd^ instar, 4 for 4^th^ instar, and 5 for adult or 5^th^ instar. We considered three alternative functions: a power function *y* = *ax*^*b*^; an exponential function y = *a*e^*bx*^; and a by-factor model where x was converted to a factor and thus log(y) was assumed to have a different mean but a constant variance for each value of x. The 5^th^ instar was grouped with the adult because most species assessed for Method 1 did not have a 5^th^ instar in their lifecycle, and for those species with a 5^th^ instar (i.e. Delphacidae), the percentage honeydew produced was similar to adult rates. Based on our compiled data, we calculated the amount of honeydew produced per hemipteran per hour, as a percentage of the body mass of each life stage. To determine the values of the parameters *a* and *b* in the first two models we used linear regression in R [64], with log-transformed y data for the exponential model and log-transformed x and y data for the power model. To determine the values of the model parameters for the third model we fitted a linear model in R with log-transformed y data and x treated as a factor, which is equivalent to a standard one-way analysis of variance (ANOVA) testing whether there was a significant difference in honeydew production with life-stage.

### Validation and case study

We validated all models for both our methods using standard leave-one-out cross validation [65]. For each model, this involved selecting one observation at a time, removing it from the data set, fitting the model to the remaining observations, using this fitted model to generate a prediction for the one removed observation, and then repeating for each observation. This resulted in a set of independent predicted values for each model. We then compared these to the observed values and calculated the mean and median percentage error.

We demonstrate the utility of the best standard method using a case study. This case study was a recalculation of the amount of honeydew produced by introduced mealybugs supporting invasion of the red imported fire ant *Solenopsis invicta* in North America [22]. Although Helms and Vinson [22] identified numerous species of bugs being tended by the ant, the pseudococcid *Antonina graminis* (Maskell) contributed 71.5% of the overall biomass of tended species and was the focus of honeydew estimation. They calculated that honeydew could supply nearly 50% of the daily energetic requirements of an *S*. *invicta* colony, with approximately 32% being produced by *A*. *graminis* alone.

## Results

### Honeydew prediction Method 1: Familial identity and body mass

The relationship between honeydew production and body mass varied greatly across hemipteran families (Table 2). Honeydew production of aphids, mealybugs (Pseudococcidae + Coccidae), and planthoppers (Delphacidae) tended to increase rapidly in the first three instars and then approached an asymptote for adults (Fig 1). For these three families, the power model was the best, with the model parameters being highly significant in each case (*p* < 0.001). For the aphids, extraordinary honeydew producers such as *M*. *fuscoviride* could be predicted using the same functional form, but just using a different intercept (Fig 1). Cross validation indicated good independent prediction accuracy for aphids (mean percentage error 26% and median percentage error 22%), mealybugs (mean percentage error 28% and median percentage error 25%) and planthoppers (mean percentage error 30% and median percentage error 28%). For leafhoppers (Cicadellidae), the best fit was achieved using an exponential model, which was highly significant (*p* < 0.001) and satisfactorily accounted for both phloem- and xylem-feeding leafhoppers, suggesting no significant difference between them (*p* = 0.40) (Fig 1). According to cross validation, independent prediction accuracy was reasonable (mean percentage error 40% and median percentage error 36%, Fig 1).

**Table 2:** Equations for the model of best fit from Method 1 for different hemipteran families (see Fig 1 and S3 Appendix) where the amount of honeydew produced (y) is a function of body mass in μg (x) for a given family.

Hemipteran group	Model of best fit	Equation
Aphididae	power-law	y = 3.46x ^0.3156^
Coccoidea	power-law	y = 0.785x ^0.672^
Delphacidae	power-law	y = 1.229x ^0.7692^
Cicadellidae	exponential	y = 24.1e ^0.001959x^
Aleyrodidae	null	y = 1.55
Psyllidae	null	y = 7.99

For both whiteflies (Aleyrodidae) and plantlice (Psyllidae) the null model was the best model, possibly indicating that honeydew production did not change with body mass, but more likely reflecting the paucity of data for these taxa (Table 2; S3 Appendix). Independent prediction error with the null model was reasonable (Aleyrodidae: mean percentage error 26% and median percentage error 25%, Psyllidae: mean percentage error 35% and median percentage error 35%) but unreliable due to the paucity of data.

### Honeydew prediction Method 2: Life stage and body mass

**Fig 2:**
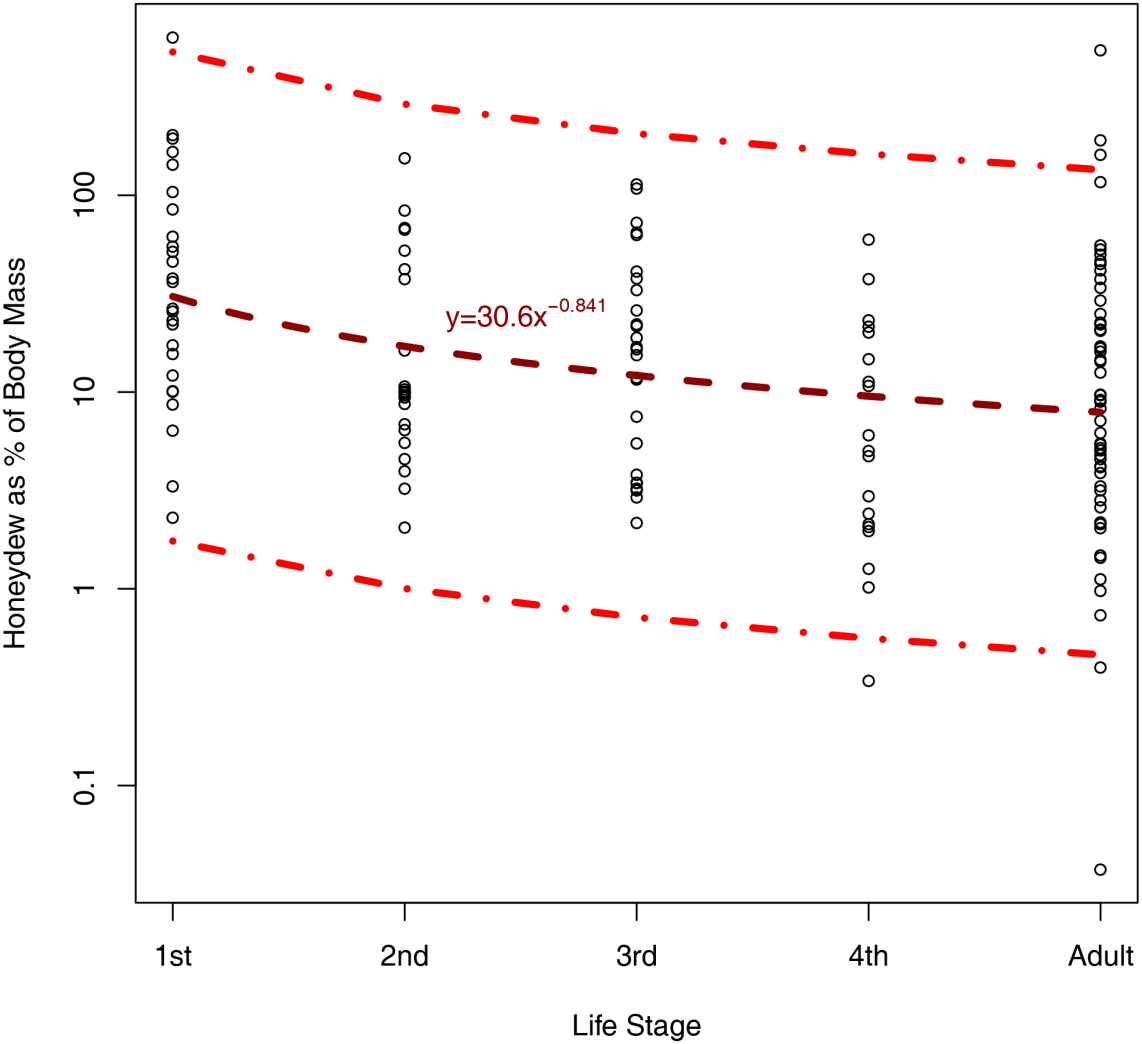
Honeydew produced as a percentage of hemipteran wet body mass (μg) per hour against life stage of Hemiptera for 21 species (see S1 Appendix), showing actual values (circles), power model prediction (dark dashed line) and 95% prediction interval (lighter dash-dotted lines).

Hemipteran families displayed a consistent pattern of rapid honeydew production as early instars, slowing with size and life stage (Fig 2). All three models (power, exponential and linear) predicting the honeydew produced as a percentage of body mass and as a function of hemipteran life stage were highly significant. This indicates that honeydew production varies with life stage and thus knowing life stage increases predictive power (*p* < 0.001 in each case). The power model y = 30.6x^-0.841^ was the best model, indicated by a lower AIC than the exponential and by-factor models (Fig 2, ΔAIC = 2.04 and 2.18, ΔAICc = 2.04 and 2.60, respectively). However, the amount of variation explained by each of the three models was low (r^2^_power_ = 0.11, r^2^_exponential_ = 0.09 and r^2^_factor_ = 0.13). Cross validation of even the best power model indicated low predictive accuracy of honeydew production for individual species (mean percentage error = 219%, median error = 184%), indicating that a species’ honeydew production must depend on many factors in addition to life stage. All hemipteran families assessed tended to produce honeydew at a rate of 10% or less of body mass per hour once adulthood was reached. Relative to their body mass, early instars had a significantly higher rate of honeydew production than later instars, despite the greater variability in production of early instars (Fig 2).

### Case study: Pseudococcids, honeydew and fire ant invasion in North America

Helms and Vinson [22] estimated the amount of honeydew from tended hemipterans *Antonina graminis* available to the invasive red imported fire ant *S*. *invicta* in North America, to demonstrate the role played by honeydew in the invasion success of the ant. Because the family of the honeydew producer is known (Pseudococcidae; Coccoidea), we re-estimated honeydew production using our most accurate method, Method 1, and compared our result with that estimated by Helms and Vinson [22] to demonstrate the utility of our method. Further details of this case study can be found in S4 Appendix.

To obtain an estimate of honeydew produced using Method 1, we used the reported body mass of *A*. *graminis* (1560 μg for adults; [22]) in our regression equation of our Coccoidea model to yield an estimate of 109.8 μg honeydew per hour, per adult. We multiplied this hourly rate by the average number of *A*. *graminis* individuals within tent shelters (10.48 ± 1.63 *A*. *graminis* per shelter: [22]) that could be tended per *S*. *invicta* colony (822.2 *A*. *graminis*: [22]) to obtain a total of 2.16 grams of honeydew per day, which is clearly much lower than the 21.6 grams per day estimated by Helms and Vinson [22]. We see two possible explanations for the difference, firstly that Helms and Vinson [22] used the aphid *Tuberolachnus salignus*, an exceptionally high honeydew producer as we have shown (see Fig 1), as their model honeydew producer. As *A*. *graminis* is a Pseudococcidae, we used our Coccoidea model, and the species in this model were all much lower producersthan *T*. *salignus*. Secondly, Helms and Vinson [22] do not state whether they included only adults or whether they counted the entire *A*. *graminis* population within shelters, which could affect the amount of honeydew produced for the entire colony (see S4 Appendix), although it is likely that it was primarily adults and 4^th^ instars (Helms pers. comm.). Thus, although we acknowledge the importance of honeydew as a critical resource, particularly in invasion mutualisms [24], our estimates suggest that *A*. *graminis* honeydew production is much lower than estimates calculated on the basis of the production rate of an unrelated hemipteran.

## Discussion

We have developed a method whereby knowing the body mass and family of a honeydew producer, a reasonably accurate estimate of honeydew production can be calculated. The best model for Cicadellidae was an exponential model, and for Aphididae, Delphacidae and Coccoidea, it was a power model. There is great potential to develop models for new families using this method as additional honeydew production data become available. Similarly, the existing family-based models could continue to be improved in the future with more data, particularly for data-deficient families from our study (i.e. Psyllidae, Aleyrodidae). If there is insufficient evidence that honeydew production changes with increases in hemipteran body mass for families, such as we found for Psyllidae and Aleyrodidae, Method 1 will provide a null (constant) model for predicting honeydew production. However, we expect that with sufficient data Method 1 will provide a model where predicted honeydew production increases with body mass.

Our second method based on body mass and life-stage was not as good at predicting honeydew production as Method 1 because the variation in honeydew production across the different families was too large. For instance, parenchyma-feeders (e.g., Diapsididae, Typhlocybinae—Cicadellidae) exude very little honeydew because of the composition of their food and their anatomy [66]. In contrast, xylem-feeders (e.g., Cercopoidea, Cicadellidae: Cicadellinae) produce huge quantities of honeydew (Fig 2d) due to the lower nutritional quality of xylem [67,68]. Without an alternative, however, Method 2 is currently the best method to use for gross estimates of honeydew production when family is unknown, which may be the case when trying to distinguish between difficult Coccoidea families, hemipteran colonies consist of species from multiple families, only nymphs are present in the colony or a familial model is lacking in method 1. We expect that future workers will be able to refine and develop method 2 to its full potential.

Honeydew production can be influenced by numerous factors, including life stage, hemipteran species, temperature, season, time of day, host plant species, density of hemipterans, levels of carbon dioxide in the atmosphere, and host plant nutrition (e.g., [25,30,31,32,36,49,69]). Tending by mutualist partners, as compared with no tending, can increase honeydew excretion rates [70], but perhaps not significantly so [34,54,71]. The identity of the partner may also be important; higher intensity of attendance by invasive ants can double honeydew production compared to native ant tending [72]. Not all of these factors were considered in our models. However, by basing our standardized method on insect body mass, the variation in production from the different factors listed above should be partly accounted for. This is because increased feeding activity under favourable conditions generally results in both a higher production of honeydew and greater body weight gain. For example, intensive feeding studies on the brown planthopper *Nilaparvata lugens* Stål have shown that when feeding on different strains of rice, feeding activity and honeydew production were significantly higher (780% and 843%, respectively) on the most susceptible variety of rice, and consequently planthoppers gained 406% more weight on the variety susceptible to the planthoppers than on the resistant rice variety [73]. Our model also does not incorporate honeydew sugar and amino acid composition, which can change depending on host plant species [70], and influence the attractiveness of honeydew for mutualists [42,74,75]. Conversely, tending by mutualists may itself change the composition of honeydew [70,71]. Dungan et al. [76] provided a simple method to estimate carbohydrate concentration within honeydew, although collection of honeydew was still required.

Our method will be invaluable to researchers who cannot identify their target taxa lower than familial level, or whose target taxa do not have published honeydew production rates and the workers themselves do not have the means to measure it directly. Identification to hemipteran family and measuring wet body mass is relatively easy, and is likely to result in a more accurate estimate of honeydew production than estimates based on an unrelated family or the production of a single other species, the only options previously available (e.g., [22]; S4 Appendix). Accurate estimates of honeydew production are immediately useful in a number of fields, such as agriculture, forestry, biological invasions, and community ecology. For example, in forestry and carbon farming our method could predict the loss of soluble carbons through honeydew secretion, which may adversely affect the economic value of tree stands [77,78,79]. In agriculture our method can be used to predict the impact of honeydew producers on crop or orchard yields by estimating the amount of honeydew produced and, consequently, the growth of microfungi (sooty mould) which is detrimental to plant growth by inhibiting photosynthetic activity. Currently the density of honeydew-producers is used as an indirect measure of the resulting sooty mould damage [80,81,82]. Our methods could also be used to predict the amount of honeydew available for honey bees in the honeydew-honey industry in South America and Europe [5,83]. Alternatively, quantifying the presence of honeydew may indicate parasitoid effectiveness, as feeding on honeydew increases parasitoid longevity and ultimately their success as biocontrol agents [84,85,86]. In invasion ecology, the colony growth and rate of spread of invasive ants is often facilitated by honeydew producers [22,23,24,25], and quantifying the amounts of honeydew available could provide more accurate information for biosecurity risk analyses and biocontrol regimes. Quantifying honeydew can give us a better understanding of food webs and nutrient fluxes in particular ecosystems [9,10,12,87]. For example, ant manure is emerging as a major driver of nutrient exchange, with the availability of honeydew both increasing ant manure rates [88] and altering the nutrients available [89]. The increased nutrient deposition may be advantageous for the plant, but then must be offset against the amount that the hemipteran is consuming from the plant [90], which can be estimated with our method. Hemiptera also contribute directly to soil nutrient loads through falling honeydew [12,13,14,15] which, again, can be estimated using our method.

## Conclusion

We have provided a quick and relatively easy method to estimate honeydew production. By providing rapid estimates of honeydew production rates without the need for laboratory trials or detailed species-specific information, it will enable faster understanding of mechanisms such as bottom-up processes (e.g., [91]) in critical systems, for example, the invasion of Christmas Island by the yellow crazy ant [27]. We expect that this method will be further improved with data from other species, particularly the Psyllidae and Aleyrodidae, for which the models did not differ significantly from the null model most likely attributable to the lack of data (S3 Appendix), and from other honeydew-producing families, especially the larger-bodied insects. However, the methods’ immediate availability for use in agriculture, invasion biology, forestry and carbon farming should prove highly beneficial for these disciplines.

## Supporting information

S1 Appendix Collation of honeydew production in different hemipteran species. (DOCX)

S2 Appendix Summary of coefficients of Hemiptera used to calculate dry mass. (DOCX)

S3 Appendix Empirical data for honeydew production per hour (on log scale), together with the model of best fit (null), for Aleyrodidae and Psyllidae. Symbols of life stage are as follows: circle—1^st^ instar nymphs, triangle—2^nd^ instar, plus symbol—3^rd^ instar, cross—4^th^ instar, diamond—5^th^ instar, upside down triangle—adults.
(PDF)

S4 Appendix Case study of pseudococcids, honeydew and fire ant invasion in North America. (DOCX)
