# Supplementary material for: Development and testing of a standardized method to estimate honeydew production
Source: PLoS One. 2018 Aug 15;13(8):e0201845. doi: 10.1371/journal.pone.0201845 (PMC6093677; doi:10.1371/journal.pone.0201845)
Supplement: S1 Appendix — (DOCX) [file pone.0201845.s001.docx]

**S1 Appendix.** Collation of honeydew production in different hemipteran species.

| **FAMILY**  ***Species*** | **Host plants** | **Species of ant tending Hemiptera** | **Instar** | **Honeydew produced** [Note: % of body mass and Log honeydew calculations not provided in literature but derived from our own calculations] | | | **References** (additional detail on data from reference provided in brackets) |
| --- | --- | --- | --- | --- | --- | --- | --- |
|  |  |  |  | **per hour (ug)** | **% of body mass** | **Log honeydew over 24 hrs** |  |
| ALEYRODIDAE |  |  |  |  |  |  |  |
| Silverleaf or Sweetpotato whitefly (*Bemisia tabaci* (Gennadius)) | Cotton (*Gossypium* sp.) |  | 1st | 2.57 | 17.36 | 1.79 | Henneberry et al. 2001 (mean values, no variation given) |
|  |  |  | 2nd | 2 | 9.95 | 1.68 |  |
|  |  |  | 3rd | 0.98 | 3.47 | 1.37 |  |
|  |  |  | 4th | 0.65 | 2.14 | 1.19 |  |
|  |  |  | Adult female | 2.66 | 5.22 | 1.80 |  |
|  |  |  |  |  |  |  |  |
| APHIDIDAE |  |  |  |  |  |  |  |
| Black bean aphid (*Aphis fabae* Scopoli) | Faba bean (*Vicia fabae* L.) | Black garden ant (*Lasius niger* (L.)) | 1st | 2.53 | 10.14 | 1.78 | Banks & Macauley 1964 (based on Tables 1, 4 and 5 as study does not provide honeydew produced per instar specifically, SE not calculated here);  Volkl et al. 1999 (from Figure 4 and Table 3); Fischer et al. 2005 (estimated from Figure 1) |
|  |  |  | 2nd | 9.64 | 10.72 | 2.36 |  |
|  |  |  | 3rd | 24.42 | 11.63 | 2.77 |  |
|  |  |  | 4th | 25.06 | 5.01 | 2.78 |  |
|  | Faba bean (*V. fabae*)*, Chenopodium album* L.*, Tanacetum vulgare* L.*, Euonymus europeaus* L.*, Cirsium arvense* (L.) |  | Adult | 42.51, 55±10, 40±8, 110±12, 118.8±10, 135.5±20, 151.5±35.5, | 4.57, 2.88, 3.17, 8.46, 9.47, 7.08, 9.13 | 3.01, 3.12, 2.98, 3.42, 3.51, 3.56, 3.45 |  |
| Cotton aphid  (*Aphis gossypii* Glover) | Cotton (*Gossypium hirsutum* L.) |  | 1st | 9.42±1.84 | 23.23 | 2.35 | Henneberry et al. 2000  Sun et al. 2009 |
|  |  |  | 2nd | 10.92±2.38 | 9.64 | 2.41 |  |
|  |  |  | 3rd | 8.23±2.67 | 3.46 | 2.29 |  |
|  |  |  | 4th | 8.94±1.88 | 1.02 | 2.33 |  |
|  |  |  | Adult | 3.71±0.72 | 0.33 | 1.95 |  |
| Cowpea aphid  (*Aphis craccivora* Koch) | Faba bean (*V. fabae*) |  | 2nd | 10.83±2.52 | 68.13 | 2.41 | Moir et al. (this study) |
|  |  |  | 3rd | 11.25±1.89 | 41.08 | 2.43 |  |
|  |  |  | 4th | 19.68±2.53 | 37.58 | 2.67 |  |
|  |  |  | Adult | 9.55±1.33 | 7.15 | 2.36 |  |
| Bluegreen or Blue alfalfra aphid (*Acyrthosiphon kondoi* Shinji.) | Clover (*Trifolium* sp.) |  | 1st | 14.22±4.42 | 55.01 | 2.53 | Moir et al. (this study) |
|  |  |  | 2nd | 28.56±4.40 | 84.03 | 2.83 |  |
|  |  |  | 3rd | 42.84±7.39 | 64.84 | 3.01 |  |
|  |  |  | Adult | 22.14±3.44 | 5.49 | 2.72 |  |
| Pea aphid (*Acyrthosiphon pisum* Harris) | *Pisum sativum* L. |  | 1st | 6.48±2.29 | 3.32 | 2.19 | Auclair 1958 |
|  |  |  | 2nd | 19.44±2.24 | 4.58 | 2.66 |  |
|  |  |  | 3rd | 33.48±4.44 | 3.45 | 2.90 |  |
|  |  |  | 4th | 66.96±10.24 | 2.96 | 3.20 |  |
|  |  |  | Adult | 87.48±11.86 | 2.14 | 3.32 |  |
| Pea aphid (*Acyrthosiphon pisum* Harris) | Faba bean (*V. fabae*) |  | 2nd | 21.87±8.10 | 11.63 | 2.65 | Moir et al. (this study) |
|  |  |  | 3rd | 37.91±7.28 | 7.43 | 2.94 |  |
|  |  |  | 4th | 33.33 | 3.26 | 2.90 |  |
|  |  |  | Adult | 61.25±3.75 | 1.60 | 3.16 |  |
| Plum thistle aphid (*Brachycaudus cardui* L.) | *T. vulgare* | Black garden ant (*L. niger*) | Adult | 190±20 | 9 | 3.66 | Volkl et al. 1999 (estimated from figure 4) |
| Turnip aphid (*Lipaphis pseudobrassicae* (Davis)) | Wild turnip (*Brassica tournefortii* Gouan.) |  | 1st | 8.09±2.95 | 8.67 | 1.85 | Moir et al. (this study) |
|  |  |  | 2nd | 15.62±4.18 | 9.41 | 2.27 |  |
|  |  |  | 3rd | 8.5±2.85 | 2.91 | 2.25 |  |
|  |  |  | 4th | 30.32±9.01 | 6.06 | 2.77 |  |
|  |  |  | Adult | 43.75±13.43 | 4.80 | 2.89 |  |
| Tansey aphid (*Macrosiphoniella tanacetaria* (Kaltenbach)) | *T. vulgare* | Black garden ant (*L. niger*) | Adult | 40±8 | 1.12 | 2.98 | Volkl et al. 1999 (estimated from figure 4) |
| Pea aphid (*Macrosiphum pisi* (Kaltenbach)) | Alfalfa (*Medicago sativa* L.) |  | 1st | 6.26±0.50 | 6.39 | 2.17 | Mittler & Sylvester 1961 (no variation given for adults) |
|  |  |  | 2nd | 23.86±1.68 | 3.97 | 2.76 |  |
|  |  |  | 3rd | 35.64±2.11 | 3.24 | 2.93 |  |
|  |  |  | 4th | 42.12±2.58 | 2.40 | 3.00 |  |
|  |  |  | Adult | 44.28 | 2.04 | 3.02 |  |
| Pink tansy aphid (*Metopeurum fuscoviride* Stroyan)*** | *T. vulgare* | Black garden ant (*L. niger*) | 1st | 250±50 | 636.13 | 3.77 | Fischer et al. 2002 (estimated from figure 1) |
|  |  |  | 2nd | 230±80 | 154.25 | 3.74 |  |
|  |  |  | 3rd | 550±120 | 108.69 | 4.12 |  |
|  |  |  | 4th | 510±140 | 59.82 | 4.08 |  |
|  |  |  | Adult | 435±80 | 29.25 | 4.02 |  |
| Rose-grain aphid (*Metopolophium dirhodum* (Walker)) | Wheat (*Triticum* sp.) |  | 1st | 15.66 | 15.66 | 2.57 | Ajayi & Dewar 1982  (estimated from figure 4, variation not estimated);  Spiller & Llewellyn 1987 (adults only) |
|  |  |  | 2nd | 16.65 | 5.55 | 2.60 |  |
|  |  |  | 3rd | 17.32 | 2.16 | 2.62 |  |
|  |  |  | 4th | 19.8 | 1.26 | 2.67 |  |
|  |  |  | Adult | 23.85,  18±1.35 | 0.98,  0.74 | 2.75,  2.63 |  |
| Onion aphid (*Neotoxoptera formosana* (Takahashi)) | Spring onion (*Allium* sp.) | Argentine ant  (*Linepithema humile* (Mayr)) | 1st | 17.92±3.99 | 10.03 | 2.63 | Moir et al. (this study) |
|  |  |  | 2nd | 20±5.40 | 6.41 | 2.68 |  |
|  |  |  | 3rd | 25.83±7.71 | 5.50 | 2.79 |  |
|  |  |  | 4th | 10.68±3.62 | 1.97 | 2.40 |  |
|  |  |  | Adult | 13.69±2.56 | 1.48 | 2.51 |  |
| Bird cherry oat aphid (*Rhopalosiphum padi* (L.)) | Wheat (*Triticum* sp.) |  | 1st | 14.37±3.57 | 143.40 | 2.53 | Moir et al. (this study);  Spiller & Llewellyn 1987 (adults only) |
|  |  |  | 2nd | 17.39±2.93 | 67.66 | 2.62 |  |
|  |  |  | 3rd | 26.80±6.32 | 72.82 | 2.80 |  |
|  |  |  | 4th | 15.41±2.15 | 14.75 | 2.56 |  |
|  |  |  | Adult | 27.96 ±4.89, 10.35 ±1.35 | 16.69,  0.85 | 2.82,  2.39 |  |
| English grain aphid (*Sitobion avenae* (Fab.)) | Wheat (*Triticum* sp.) |  | 1st | 14.85 | 37.78 | 2.55 | Ajayi & Dewar 1982 (estimated from figure 4, variation not estimated) |
|  |  |  | 2nd | 15.03 | 10.08 | 2.55 |  |
|  |  |  | 3rd | 16.06 | 3.17 | 2.58 |  |
|  |  |  | 4th | 17.55 | 2.058 | 2.62 |  |
|  |  |  | Adult | 20.20 | 1.44 | 2.68 |  |
| Spotted alfalfa aphid (*Therioaphis maculata* (Buckton)) | Alfalfa (*M. sativa*) |  | 1st | 3.78±0.38 | 12.19 | 1.95 | Mittler & Sylvester 1961 (no variation given for adults) |
|  |  |  | 2nd | 6.48±0.55 | 9.97 | 2.19 |  |
|  |  |  | 3rd | 23.76±0.48 | 11.88 | 2.75 |  |
|  |  |  | 4th | 33.48±2.47 | 10.8 | 2.90 |  |
|  |  |  | Adult | 44.71 | 9.63 | 3.03 |  |
| Black citrus or tea aphid (*Toxoptera aurantii* (Boyer de Fonscolombe)) | Tea (*Camellia sinensis* (L.) Kuntze) |  | 1st | 14.08±0.17 | 2.31 | 2.53 | Baoyu & Chengsong  2007 (variation units not defined, assuming SD) |
|  |  |  | 2nd | 26.81±0.92 | 3.23 | 2.81 |  |
|  |  |  | 3rd | 47.71±0.76 | 3.80 | 3.06 |  |
|  |  |  | 4th | 74.07±1.64 | 4.73 | 3.25 |  |
|  |  |  | Adult | 53.27±1.39 | 2.18 | 3.11 |  |
| Giant willow aphid (*Tuberolachnus salignus* (Gmelin))*** | Willow (*Salix alba* L. and *S. acutifolia* Willd.*)* |  | 1st | 416.67, 486 | 166.67, 194.4 | 4, 4.06 | Mittler 1957, 1958 (variation not given); Hargraves & Llewellyn 1987 (taken from Figure 2, variation not extracted) |
|  |  |  | 2nd | 562.5, 788.4 | 37.5, 52.56 | 4.13, 4.27 |  |
|  |  |  | 3rd | 1015.2, 1291.67 | 33.12, 26.03 | 4.38, 4. 49 |  |
|  |  |  | 4th | 1512, 1625 | 21.6, 23.21 | 4.55, 4.59 |  |
|  |  |  | Adult | 750, 2048.4 | 5.41, 17.07 | 4.35, 4.69 |  |
| Brown sow-thistle aphid (*Uroleucon sonchi* (L.)) | Sow thistle (*Sonchus oleraceus* L.) |  | 1st | 50.20±18.20 | 203.01 | 3.08 | Moir et al. (this study) |
|  |  |  | 2nd | 56.48±8.16 | 16.27 | 3.13 |  |
|  |  |  | 3rd | 46.44±9.34 | 7.51 | 3.04 |  |
|  |  |  | 4th | 106.09±32.69 | 11.28 | 3.40 |  |
|  |  |  | Adult | 149.33±30.43 | 4.16 | 3.55 |  |
|  |  |  |  |  |  |  |  |
| CICADELLIDAE |  |  |  |  |  |  |  |
| Maize leafhopper (*Cicadulina mbila* Naudé) | Maize (*Zea* *mays* L.) |  | Adult | 41.25 | 8.25 | 2.99 | Mesfin et al 1995 (from Table 1, no variation given) |
|  | *Digitaria sanguinalis* (L.) Scop. |  | Adult | 254.58 | 46.45 | 3.78 |  |
|  | sugar cane (*Saccharum officinarum* L.) |  | Adult | 210 | 20.58 | 3.70 |  |
|  | *Zea perennis* (Hitchc.) Reeves & Mangelsd. |  | Adult | 43.75 | 4.20 | 3.02 |  |
| Rice green leafhopper (*Nephotettix cincticeps* Uhler) | Rice (*Oryza* sp.) |  | Adult | 1041.67, 3744 | 44.70, 160.68 | 4.39, 4.95 | Oya 1980 (SD or SE not given); Kawabe 1985 (no variation given) |
| Green rice leafhopper (*Nephotettix virescens* (Distant)) | Rice (*Oryza* sp.) |  | 1st | 40.25±5.89 | 46.22 | 2.98 | Foissac et al 2000 (from Table 1) |
|  |  |  | 5th | 306.81±82.47 | 14.61 | 3.86 |  |
| Gamagrass leafhopper (*Dalbulus quinquenotatus* DeLong & Nault)* | *Tripsacum pilosum* Scribn. & Merr. | Pavement ant (*Tetramorium caespitum* (L.)) | Adult | 16203.7±1851.85 | 548.5 | 5.59 | Larsen et al. 1992 (from Figure 2) |
| Corn leafhopper (*Dalbulus maidis* (DeLong & Wolcott))* | Sweet corn (*Z. mays* convar. *saccharata* var. *rugosa*) |  | Adult | 4629.63±1388.89 | 191 | 5.04 |  |
| *Dalbulus gelbus* DeLong* | Sweet corn (*Z. mays* convar. *saccharata* var. *rugosa*) |  | Adult | 2314.81±925.95 | 117.14 | 4.74 |  |
|  |  |  |  |  |  |  |  |
| COCCIDAE |  |  |  |  |  |  |  |
| Brown soft scale (*Coccus hesperidum* L) | *Ficus benjamina* L. |  | 1st | 3.32 | 61.93 | 1.90 | Gollan 2010 (variation not given) |
|  |  |  | 3rd | 94 | 37.97 | 3.35 |  |
|  |  |  | Adult | 284 | 9.70 | 3.83 |  |
|  |  |  |  |  |  |  |  |
| DELPHACIDAE |  |  |  |  |  |  |  |
| Brown planthopper (*Nilaparvata lugens* Stål) | Rice (*Oryza* sp.) |  | 1st | 22.33±4.71 | 25.64 | 2.72 | Foissac et al 2000 (1^st^ and 5^th^ instars); Qiu et al 2011 (3^rd^ instar, values estimated from Figure 2);  Yang et al. 2012 (5^th^ instar, values from Figure 3, SE not extracted);  Wada et al 2008 (adults that were 1-5 days old) |
|  |  |  | 3rd | 138.66±10, 403±41, 725.83±140 | 21.67, 62.96, 113.41 | 3.98, 3.52, 4.24 |  |
|  |  |  | 5th | 59.4±22.58,  1333.33, 416.67, 833.33, 416.67, 750, 208.33 | 16.02, 50, 20.83, 12.62, 41.67, 53.33, 2.82, | 4.50,  4, 4.30, 4.25, 4, 3.69, 3.15 |  |
|  |  |  | Adult | 208.33±1.59, 333.33±2.03, 583.33±4.69, 875±3.71, 791.67±1.52 | 33.97, 37.55, 25.03, 14.30, 8.94 | 4.27, 4.32, 4.14, 3.90, 3.69 |  |
| White-backed planthopper (*Sogatella furcifera* (Horváth)) | Rice (*Oryza* sp.) |  | 1st | 16.95 | 84.78 | 2.60 | Zhu & Cheng 2002  (given as both dry mass weight and mm^2^ of honeydew excreted. No variation provided) |
|  |  |  | 2nd | 33.72 | 42.15 | 2.90 |  |
|  |  |  | 3rd | 59.59 | 17.02 | 3.15 |  |
|  |  |  | 4th | 96.76 | 20.15 | 3.36 |  |
|  |  |  | 5th | 138.10 | 22.63 | 3.52 |  |
|  |  |  | Adult | 354.70 | 22.16 | 3.93 |  |
|  |  |  |  |  |  |  |  |
| MEMBRACIDAE |  |  |  |  |  |  |  |
| *Guayaquila xiphias* (Fab.) | *Didymopanax vinosum* (Cham. & Schltdl.) Seem. | *Camponotus crassus* Mayr*, C. rufipes* (Fab.)*, C. atriceps* (Smith) [listed incorrectly *C. abdominalis* (F.)] *, C. renggeri* Emery*, C.* aff. *Blandus* (Smith) | Adult | 7833.33 ±1333.33  13500±3166.67 | 28.36  38.16 | 5.27  5.51 | Quental et al. 2005  (number of droplets produced. All droplets ~ 0.7mm diameter.) |
|  |  |  |  |  |  |  |  |
| PSEUDOCOCCIDAE |  |  |  |  |  |  |  |
| Cotton mealybug (*Phenacoccus solenopsis* Tinsley) | Tomato (*Solanum lycopersicum* L.) | Red imported fire ant (*Solenopsis invicta* Buren) | 1st | 4.54 ±0.12 | 51.84 | \| 2.03 \| \| --- \| | Zhou et al. 2013 |
|  |  |  | 2nd | 11.64±0.74 | 10.30 | 2.44 |  |
|  |  |  | 3rd | 95.10±5.83 | 18.91 | 3.35 |  |
|  |  |  | Adult | 169.08±9.26 | 3.89 | \| 3.60 \| \| --- \| |  |
| *P. solenopsis* | *Hibiscus rosa-sinensis* L. | Red imported fire ant  (*S. invicta*) | 1st | 2.98±0.10 | 36.39 | 1.85 |  |
|  |  |  | 2nd | 8.92±0.51 | 8.73 | 2.33 |  |
|  |  |  | 3rd | 81.40±1.84 | 16.48 | 3.29 |  |
|  |  |  | Adult | 126.56±5.91 | 3.32 | 3.48 |  |
| *P. solenopsis* | Cotton  (*Gossypium* sp.) | Red imported fire ant  (*S. invicta*) | 1st | 2.3±0.12 | 26.5896 | 1.74 |  |
|  |  |  | 2nd | 7.53±0.83 | 6.86 | 2.25 |  |
|  |  |  | 3rd | 74.89±2.81 | 15.41 | 3.25 |  |
|  |  |  | Adult | 113.51±2.87 | 3.17 | 3.43 |  |
| Gray pineapple mealybug (*Dysmicoccus neobrevipes* Beardsley) | *Yucca* sp. |  | 1st | 13.33 | 22.07 | 2.50 | Nishida & Kuramoto 1963 (taken from Figure 3, SE not extracted) |
|  |  |  | 2nd | 3.12 | 2.05 | 1.87 |  |
|  |  |  | 4th | 5.41 | 0.34 | 2.11 |  |
|  |  |  | Adult | 0.83 | 0.03 | 1.30 |  |
| Vine mealybug (*Planococcus ficus* (Signoret)) | Grape vine (*Vitis Vinifera* L.) | Argentine ant  (*L. humile*) | 2nd | 1.18±0.38 | 78.61 | 1.45 | Choi et al. 2009 (taken from Figure 1) |
| Obscure, Californian or Tuber mealybug (*Pseudococcus viburni* Signoret) | Grape vine (*V. vinifera*) |  | 2nd | 1.16±0.42 | 77.5 | 1.44 |  |
| Long-tailed mealybug (*Pseudococcus longispinus* (Targioni Tozzetti)) | Grape vine (*V. vinifera*) |  | 2nd | 0.72±0.19 | 48.05 | 1.24 |  |
| PSYLLIDAE |  |  |  |  |  |  |  |
| Plant louse (*Acizzia* sp. nov.) | *Acacia saligna* (Labill.) H.L.Wendl. | Argentine ant  (*L. humile*) | 1st | 3.12 | 104.16 | 1.87 | Moir et al. (this study) |
|  |  |  | 2nd | 20.55±4.61 | 67.16 | 2.69 |  |
|  |  |  | 3rd | 5.20 | 22.16 | 2.09 |  |
|  |  |  | 4th |  |  |  |  |
|  |  |  | Adult | 12.18±4.38 | 5.04 | 2.46 |  |

*Renowned high producers of honeydew
