# Supplementary material for: Development and testing of a standardized method to estimate honeydew production
Source: PLoS One. 2018 Aug 15;13(8):e0201845. doi: 10.1371/journal.pone.0201845 (PMC6093677; doi:10.1371/journal.pone.0201845)
Supplement: S2 Appendix — (DOCX) [file pone.0201845.s002.docx]

**S2 Appendix.** Summary of coefficients of Hemiptera used to calculate dry mass. Families in bold are those presented in Gruner [61], while families not in bold do not have published information on their specific coefficients and are therefore listed with their closest phylogenetic families (following [92]).

| **Family** | **a** | **b** |
| --- | --- | --- |
| **Aphididae**, Pseudococcidae, Coccidae, Margarodidae, Aleyrodidae | 0.0175 | 2.629 |
| **Cicadellidae**, Membracidae | 0.0067 | 3.141 |
| **Delphacidae** | 0.0206 | 2.764 |

92. Cryan JR, Urban JM. Higher-level phylogeny of the insect order Hemiptera: Is Auchenorrhyncha really paraphyletic? Systematic Entomology. 2012; 37: 7-21.
