# Supplementary figures and images for: Development and testing of a standardized method to estimate honeydew production
Source: PLoS One. 2018 Aug 15;13(8):e0201845. doi: 10.1371/journal.pone.0201845 (PMC6093677; doi:10.1371/journal.pone.0201845)

## Aleyrodidae

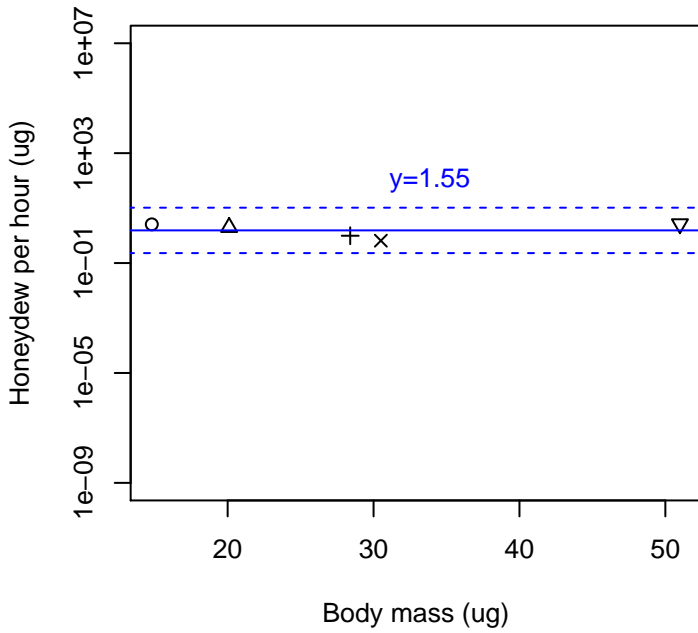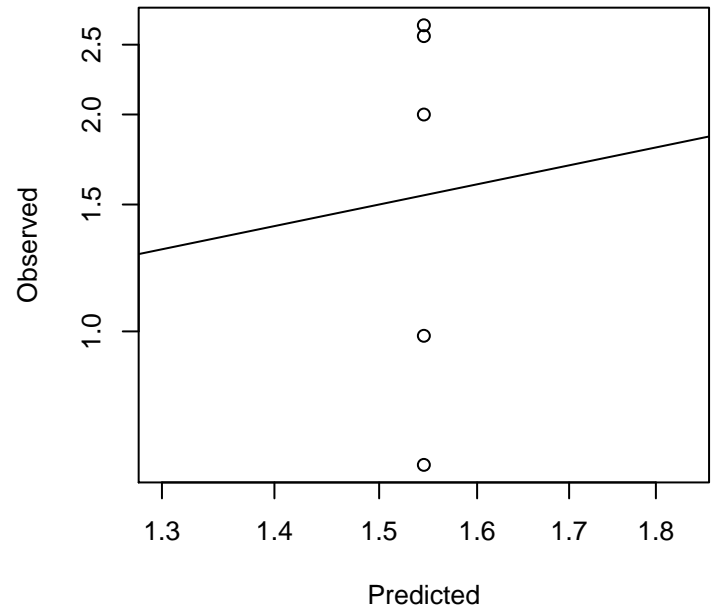

## Psyllidae

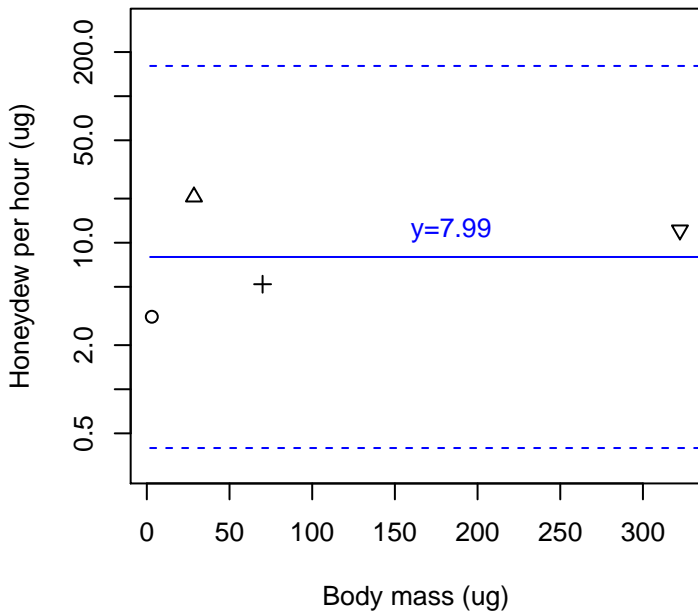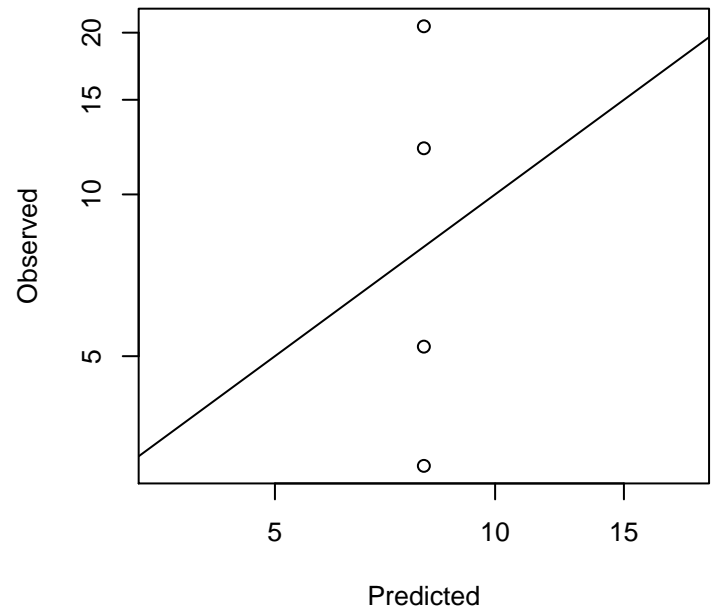

Supplement: S3 Appendix — Symbols of life stage are as follows: circle—1st instar nymphs, triangle—2nd instar, plus symbol—3rd instar, cross—4th instar, diamond—5th instar, upside down triangle—adults. (PDF) [file pone.0201845.s003.pdf]
