# Supplementary material for: Development and testing of a standardized method to estimate honeydew production
Source: PLoS One. 2018 Aug 15;13(8):e0201845. doi: 10.1371/journal.pone.0201845 (PMC6093677; doi:10.1371/journal.pone.0201845)
Supplement: S4 Appendix — (DOCX) [file pone.0201845.s004.docx]

**S4 Appendix.** Case study of Pseudococcids, honeydew and fire ant invasion in North America

Helms and Vinson [22] estimated the amount of honeydew from tended hemipterans available to the invasive red imported fire ant *Solenopsis invicta* in North America, to demonstrate the role played by honeydew in the invasion success of the ant. Although Helms and Vinson [22] identified numerous species of bugs being tended by the ant, the pseudococcid *Antonina graminis* (Maskell) contributed 71.5 % of the overall biomass of tended species and was, therefore, the focus of honeydew estimation. They calculated that honeydew could supply nearly 50 % of the daily energetic requirements of an *S. invicta* colony, with approximately 32 % being produced by *A. graminis* alone. As the family of the honeydew producer is known (Pseudococcidae; Coccoidea), we estimated honeydew production with Method 1 which was considered the most accurate method, and compare our result with that estimated by Helms and Vinson [22].

To obtain an estimate of honeydew produced using Method 1, we used the reported body mass of *A. graminis* (1560 μg for adults: [22]) in our regression equation to yield an estimate of 109.8 µg honeydew per hour, per adult. We multiplied this hourly rate by the average number of *A. graminis* individuals within tent shelters (10.48 ± 1.63 *A. graminis* per shelter: [22]) that could be tended per *S. invicta* colony (822.2 *A. graminis*: Helms & Vinson 2002) to obtain a total of 2.16 grams of honeydew per day, which is clearly much lower than the 21.6 grams per day estimated by Helms and Vinson (2002).We see two possible explanations for the difference, firstly that Helms and Vinson [22] used a very high producing aphid as their model honeydew producer compared with the lower producing Coccoidea that we use, and we discuss this further below. Secondly, Helms and Vinson [22] do not state whether they included only adults or whether they counted the entire *A. graminis* population within shelters. We examine how this could alter our results below.

It is very difficult to infer how many individuals of each life stage might have been present within *A. graminis* colonies because this will change depending on the season and other factors such as the presence of parasitoids and predators. We consider three scenarios; 1. that each life stage was represented equally in colonies with the total population being 822, 2. that each life stage was represented equally in colonies but that 822 represented only adults and the total number of *A. graminis* including nymphs was approximately 4110 (822*5), and 3. that the life stages were not equally represented in the colony, and that 822 were predominantly the larger adults and 4^th^ instars, with few younger instars present. A fourth scenario, that life stages were unequally represented but the majority were the smaller 1^st^, 2^nd^, and/or 3^rd^ instars, is not supported by Helms and Vinson [22], as they record individuals of *A. graminis* weighing 1.56 mg, which is equivalent to 4^th^ instars and adults of other Pseudococcidae species (see Appendix A). In scenario 1 the amount of honeydew available per day from each shelter would be 0.74 g, in scenario 2 the honeydew produced would be 3.69 g per day, and in scenario 3 with only adults present, 2.16 g per day would be available. All are lower than Helms and Vinson’s estimate of 21.6 g.

We do not dispute that honeydew is an important resource for *S. invicta*, as has been shown in multiple subsequent studies (Helms et al. 2011; Wilder et al. 2011; Zhou et al. 2014). However, our method, which is based on the honeydew production of three species in the same family instead of an unusually high producer in a different family, calls into question whether *A. graminis* alone provides such a large proportion of the energy requirements of *S. invicta*. If Helms and Vinson [22] did overestimate the honeydew production then it was most likely because they based *A. graminis* honeydew production on that of *Tuberolachnus salignus* (Aphididae), an exceptionally high honeydew producer (see Fig. 1). It is possible that *A. graminis* is also an exceptionally high honeydew producer, although no equivalent aphids, nor any other mealybug species that we could find, produce the same rates. As Helms and Vinson point out, their estimate is heavily reliant on how closely the honeydew production of *A. graminis* follows that of *T. salignus*. We acknowledge that our rate appears low in comparison to Helms and Vinson’s estimate, but it is possible that the ants were obtaining additional daily energy from harvesting the Hemiptera themselves, in addition to their honeydew, or other prey. Alternatively, it is possible that *A. graminis* is an exceptionally high honeydew producer, or that *S. invicta* is capable of harvesting more honeydew than other ants [93]. However, our standard rate for mealybugs included the grapevine pest *Planococcus ficus,* which is tended by the introduced Argentine ant, *Linepithema humile* [34], and the cotton pest *P. solenopsis*, which is also tended by *S. invicta* [93] and, therefore, we think it unlikely that *A. graminis* produces higher quantities than these other ant-tended invasive mealybugs. Another possibility is that other hemipteran species could have been providing more honeydew than *A. graminis* even though they were not as abundant*.* [94] have demonstrated that *S. invicta* harvest significantly more honeydew from colonies of aphids than mealybugs, particularly over longer time periods. *Solenopsis invicta* may thus have been meeting its daily energy requirements through aphid-tending, in addition to pseudococcid-tending.

Honeydew availability and variability may be primarily determined by hemipteran density. Choi [34] noted that invasive hemipterans do not produce more honeydew than individuals of other species, but have higher reproductive rates with more generations per year and are able to attain higher abundances. Beggs *et al.* [39] also found that honeydew variability across host plants and season was a function of hemipteran density, with higher densities producing more honeydew. Despite Helms and Vinson [22] counting individual *A. graminis* in colonies, they did not specify that instars were included in the mean estimate of 822 individuals per colony, only giving weights for adults. Thus, *A. graminis* may have occurred in higher densities than were reported, as we noted by considering three different scenarios. Although we acknowledge the importance of honeydew as a critical resource, particularly in invasion mutualisms [24], our estimates suggest that *A. graminis* honeydew may not be providing the majority of the energy for the fire ant invasion in the study of Helms and Vinson [22]. Indeed, more recently Helms [95] suggested that the importance of honeydew is not that it supplies an inordinate amount of the energy budget of ant colonies, but rather that the carbohydrates in honeydew is often limited and are essential in fuelling the activities of workers. Helms and Vinson [22] provided an innovative and fundamental study into invasive ants and the resources that support them; however, further study on all members of the mutualism guild that fire ants tend in this system is warranted to determine their specific contributions to the energy requirements of the fire ant.

93. Zhou A, Lu Y, Zeng L, Xu Y, Liang G. Does mutualism drive the invasion of two alien species? The case of *Solenopsis invicta* and *Phenacoccus solenopsis.* PloS ONE. 2012a; 7: e41856.

94. Zhou A, Lu Y, Zeng L, Xu Y, Liang G. Fire ant-hemipteran mutualisms: Comparison of ant preference for honeydew excreted by an invasive mealybug and a native aphid. Sociobiology. 2012b; 59: 795-804.

# 95. Helms KR. Mutualisms between ants (Hymenoptera: Formicidae) and honeydew-producing insects: Are they important in ant invasions? Myrmecological News. 2013; 18: 61-71.
